# The impact of early target attainment of vancomycin in critically ill patients with confirmed Gram-positive infection: A retrospective cohort study

**DOI:** 10.1186/s12879-021-06840-y

**Published:** 2021-11-24

**Authors:** Khalid Al Sulaiman, Abdulrahman Alshaya, Ohoud Aljuhani, Amjad Alsaeed, Nadiyah Alshehri, Ramesh Vishwakarma, Hamdan Alzahrani, Sara Althewaibi, Nawaf Alghamdi, Khalid Alhelal, Aisha Alharbi, Shmeylan Al Harbi

**Affiliations:** 1 Pharmaceutical Care Department, King Abdulaziz Medical City, Riyadh, Saudi Arabia; 2 College of Pharmacy, King Saud Bin Abdulaziz University for Health Sciences, Riyadh, Saudi Arabia; 3 King Abdullah International Medical Research Center, Riyadh, Saudi Arabia; 4 Department of Pharmacy Practice, Faculty of Pharmacy, King Abdulaziz University, Jeddah, Saudi Arabia; 5 Statistics Department, European Organization for Research and Treatment of Cancer (EORTC) Headquarters, Brussels, Belgium; 6 Microbiology Department, King Abdulaziz Medical City, Riyadh, Saudi Arabia; 7 Pharmaceutical Care Department, King Abdulaziz University Hospital, Jeddah, Saudi Arabia; 8 King Abdulaziz Medical City, King Abdullah International Medical Research Center/King Saud bin Abdulaziz University for Health Sciences, Riyadh, Saudi Arabia

**Keywords:** Vancomycin, Trough level, Mortality, Infection, Critical care, Intensive care unit

## Abstract

**Background:**

Vancomycin is a commonly used antibiotic in critically ill patients for various indications. Critical illness imposes pharmacokinetic-pharmacodynamics challenges, which makes optimizing vancomycin in this population cumbersome. Data are scarce on the clinical impact of time to therapeutic trough levels of vancomycin in critically ill patients.  This study aims to evaluate the timing to achieve therapeutic trough level of vancomycin on 30-day mortality in critically ill patients.

**Method:**

A retrospective cohort study was conducted for all adult critically ill patients with confirmed Gram-positive infection who received IV vancomycin between January 1, 2017, and December 31, 2020. We compared early (< 48 h) versus late (≥ 48 h) attainment of vancomycin therapeutic trough levels. The primary outcome was the 30-day mortality in critically ill patients. Secondary outcomes were the development of resistant organisms, microorganisms eradication within 4–5 days of vancomycin initiation, acute kidney injury (AKI), and length of stay (LOS). Propensity score-matched (1:1 ratio) used based on patient’s age, serum creatinine, and albumin values at baseline.

**Results:**

A total of 326 patients were included; 110 patients attained the therapeutic trough levels within 48 h of vancomycin initiation. Late achievement of the therapeutic trough levels was associated with higher 30-day mortality (HR: 2.54; 95% CI [1.24–5.22]; p = 0.01). Additionally, patients who achieved therapeutic trough levels of vancomycin late were more likely to develop AKI (OR = 2.59; 95% CI [1.01–6.65]; p = 0.04). Other outcomes were not statistically significant between the two groups.

**Conclusion:**

Early achievement of vancomycin therapeutic levels in patients with confirmed Gram-positive infection was associated with possible survival benefits.

## Background

Infections may induce sepsis or septic shock, which is common in critically ill patients [1]. Gram-positive infections are a growing concern given the increase in their resistant patterns, including methicillin-resistant Staphylococcus aureus (MRSA) with a reported mortality rate up to 55% in critically ill patients [2, 3]. Vancomycin is still commonly used for suspected or confirmed Gram-positive infections in critically ill patients, despite having newer antimicrobial therapies with MRSA coverage [3–8]. Vancomycin dosing and monitoring in critically ill patients is still challenging, despite being in the market for over 60 years [6–8].

Vancomycin requires a deep understanding of its pharmacokinetic-pharmacodynamic (Pk-PD) properties in various patient populations, and due to its narrow therapeutic index, vancomycin requires frequent therapeutic drug monitoring (TDM) to determine its safety and efficacy [4, 9]. Critical illness may significantly impact patients' volume of distribution, metabolism, and excretion, which adds another hurdle in promptly achieving therapeutic levels [9]. Several factors are associated with failure to achieve initial therapeutic vancomycin trough levels in critically ill patients include male gender, young patients, omission of the loading dose, augmented renal clearance (ARC), and high albumin levels [3–5]. The recent recommendation suggested that the area under the curve (AUC)-guided vancomycin monitoring strategy should be utilized in patients with MRSA infections due to superiority in efficacy as well as nephrotoxicity data [7, 8]. However, due to the complexity of AUC-guided vancomycin monitoring in clinical practice, the vancomycin trough level remains the most common and practical method for monitoring vancomycin efficacy and safety [8].

Vancomycin-induced acute kidney injury remains the most common adverse drug reaction (ADR) with the current TDM strategy that carries an increased risk for prolonged hospitalization [3, 4, 7, 8]. The fear of this ADR may cause some practitioners to be hesitant in prescribing the optimal vancomycin dosing, which may lead to emerging of  more resistant organisms [3, 5]. The consensus of the American society of health-system pharmacists (ASHP), the infectious diseases society of America (IDSA), and the society of infectious diseases pharmacists (SIDP) provide recommendation for the appropriate timing to check vancomycin trough levels [7, 8]. They suggested to draw vancomycin trough level immediately before the fourth dose, which would be within two days per the previous TDM.

According to our knowledge, no studies evaluated the correlation between the time to achieve the therapeutic trough level and critically ill patients' clinical outcomes. Therefore, we aimed to study the correlation of early achievements of therapeutic trough levels of vancomycin on 30-day mortality in critically ill patients.

## Methods

### Study design

A retrospective cohort study of critically ill patients admitted to intensive care units (ICUs) with confirmed Gram-positive infections (e.g., MSSA, MRSA) who received intravenous vancomycin (weight-based dosing). All patients who met our inclusion criteria during the study period from January 01, 2017 to December 31, 2020 were included. Patients were categorized into two groups based on the timing of achieving therapeutic vancomycin trough level during their ICU stay to an early and late group. We defined the early group as achieving therapeutic vancomycin trough levels (15–20 mg/L (or 10–14 μmol/L)) within 48 h of the first intravenous vancomycin exposure. Vancomycin trough levels were obtained after reaching the steady-state either 30 min before the fourth dose (without a loading dose) or before the third dose (with loading dose). Critical care pharmacists were responsible for vancomycin TDM in their respected critical care units. No specific nomogram was followed. The study was approved by King Abdullah International Medical Research Center (KAIMRC)-Institutional Review Board, Riyadh, Saudi Arabia (Reference No: RC20/587/R).

Gram stain is used to differentiate between Gram-positive and negative bacteria. Blood and MacConkey agar are used to culture microorganisms; after 24 h of incubation, a single colony is selected and smeared directly as a thin film on a Matrix-assisted laser desorption/ionization-time of flight (MALDI-TOF) biomerieux for pathogen identification, then VITEKR 2 is used thereafter to determine susceptibility.

Bacteria were identified in the blood, urine, wound, drainage, cerebrospinal fluid (CSF), and respiratory specimens. Confirmed infection defined as sputum or endotracheal aspiration shows growth ≥ of 100,000 CFU/ml; Bronchoalveolar lavage (BAL) shows growth ≥ of 10,000 CFU of single organism/ml for protected specimen brushes (PSBs), and ≥ 100,000 CFU of single organism/ml for BAL fluid. Additionally, urinary cultures were considered significant if showing a growth of ≥ 100,000 CFU/ml of no more than two species of microorganisms [29]. Cultures were excluded if the laboratory reported them as a "contaminant sample."

### Eligibility criteria

**Fig. 1:**
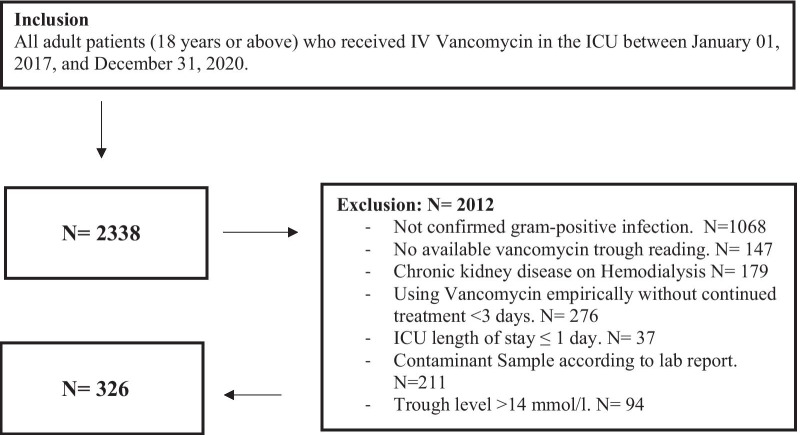
Flow diagram of inclusion/exclusion criteria, and for eligible patients who underwent analysis

Patients were enrolled in the study if they were critically ill, aged 18 years or older with confirmed Gram-positive infection, and received IV vancomycin between January 01, 2017 to December 31, 2020. Exclusion criteria include using vancomycin empirically without continued treatment (Duration < 3 days) or no available vancomycin trough reading. Besides, patients with CKD on hemodialysis (HD), have an initial trough level > 14 μmol/L, contaminant sample, ICU LOS ≤ one day or labeled as "Do-Not-Resuscitate" status within the first 24 h of ICU admission were excluded (Fig. 1).

### Setting

This study was conducted in the adult medical, surgical, trauma, and burn ICUs at King Abdulaziz Medical City (KAMC), a tertiary-care academic referral hospital in Riyadh, Saudi Arabia. The ICU admits medical, surgical, trauma, burn patients and operates as closed units with 71 ICU beds capacity with 24/7 onsite coverage by critical care board-certified intensivists and clinical pharmacists.

### Data collection

Demographic data, Acute Physiology And Chronic Health Evaluation II (APACHE II) score, Modified Sequential Organ Failure Assessment (mSOFA), comorbidities, laboratory tests, cultures (Blood, Skin, Respiratory, Urine, CSF), microorganism (s), vancomycin date of administration, vancomycin initial trough concentrations, time to reach the therapeutic levels, development of resistant organisms (e.g., Vancomycin Intermediate Staphylococcus Aureus (VISA), Vancomycin-Resistant Staphylococcus Aureus (VRSA) or Vancomycin-Resistant Enterococcus (VRE)), and vancomycin induced nephrotoxicity (VIN) were collected from an electronic record system (Best Care system). All variables have been compiled in an electronic data collection sheet. Patients were followed during ICU stay until death or discharge, whichever occurred first.

### Endpoint (s)

The primary endpoint evaluated the association between the timing of achieving therapeutic levels of vancomycin (early vs. late) in critically ill patients and mortality at 30 days from admission. Secondary endpoints include developing a vancomycin-resistant organism, microorganisms eradication within 4–5 days of vancomycin initiation, vancomycin-induced nephrotoxicity, and LOS.

### Definition(s)/procedure(s)

1. Critically ill patient was defined as patient who is admitted in the intensive care unit (s) because of life threatening or potential life-threatening physiological alterations requiring intense and vigilant monitoring and medical care.
2. The 30-day mortality was defined as the in-hospital death occurring for any cause within 30 days of the admission date during the hospital stay.
3. Acute kidney injury (AKI) was defined using Acute Kidney Injury Network (AKIN) definition, which is a sudden decrease of renal function within 48 h, defined by an increase in absolute SCr of at least 26.5 μmol/L (0.3 mg/dL) or an increase in SCr ≥ 50% from baseline (1.5 × baseline value) [10].
4. Susceptibility of Gram-positive bacteria based on Clinical Laboratory Standards Institute (CLSI) [11]:
  Methicillin-Sensitive Staphylococcus Aureus (MSSA): Clinical isolate of *Staphylococcus aureus* sensitive to oxacillin; Minimal inhibitory concentration (MIC) < 2 μg/mL.
  Methicillin-Resistant Staphylococcus Aureus (MRSA): Clinical isolate of *Staphylococcus aureus* resistant to all beta-lactams except Ceftaroline*; *MIC  < 4 μg/mL.
  Vancomycin Intermediate resistant *Staphylococcus aureus* (VISA): Clinical isolate of *Staphylococcus aureus* with intermediate susceptibility to vancomycin; MIC 4–8 μg/mL.
  Vancomycin-resistant *Staphylococcus aureus* (VRSA): Clinical isolate of *Staphylococcus aureus* that is resistant to vancomycin; MIC > 8 μg/mL.
  Vancomycin-Resistant Enterococcus (VRE): Clinical isolate of Enterococcus resistant to vancomycin; MIC > 8 μg/mL.

### Data management and statistical analysis

Categorical variables were reported using numbers and percentages, while continuous variables were reported using mean with standard deviation (SD) or median with interquartile range (IQR) when appropriate. We compared normally distributed numerical variables with the t-test and other continuous variables with the Mann–Whitney U test. In addition, we compared categorical variables using the Chi-square or Fisher exact test. The normality assumptions were assessed for all continuous variables using graphical representation (i.e., histograms and Q-Q plots) and statistical tests (i.e., Shapiro–Wilk test). The baseline and clinical characteristics were compared between the early and late initiation groups. No imputation was made for missing data as the cohort of patients in our study was not derived from random selection.

Propensity score matching Procedures (Proc PS match) (SAS, Cary, NC) was used to match patients (1:1 ratio) who achieved early therapeutic vancomycin level to patients who did not, based on the patient’s age, serum creatinine, and albumin levels. Clinically relevant variables were included in the model if they differed between study groups and were associated with the primary outcome. A greedy nearest neighbor matching method was used in which one patient in the late group is matched with one patient in the early group (control), which eventually produced the smallest within-pair difference among all available pairs with treated patients. Patients were matched only if the difference in the logits of the propensity scores for pairs of patients from the two groups was less than or equal to 0.5 times the pooled estimate of the standard deviation.

Multivariable Cox proportional hazards regression analyses were performed for the 30-day mortality. For the other outcomes considered in this study, multivariable and negative binomial regression analyses were used as appropriate. Regression analysis was done by considering the PS score as one of the covariates in the model. The odds ratios (OR), hazard ratio (HR), or estimates with the 95% confidence intervals (CI) were reported as appropriate. We considered a P value of < 0.05 statistically significant and used SAS version 9.4 for all statistical analyses.

### Sample size calculation

The sample size was calculated using Power Analysis and Sample Size (PASS) software (PASS 15 Power Analysis and Sample Size Software (2017). From a pilot study of 30 patients, the ICU mortality was estimated to be 25% in the late group, and we were expecting a reduction in 30-day mortality by 13.3% in the early group (i.e., 11.7%). With 80% power to detect a difference in the 30-day mortality between the two groups of 13.3% and one-sided Z-test statistics with pooled variance. A total sample size of 209 was considered to assess the study's primary endpoint.

## Results

### Result section

A total of 2338 critically ill patients were admitted to the ICUs during the study period; 326 patients were eligible for inclusion. Before PS matching, 110 patients (33.7%) attained the therapeutic trough levels within 48 h of vancomycin initiation compared to 216 patients who late achieved the trough level. According to the selected criteria, a total of 210 patients were matched using propensity score matching (1:1). Among 210 patients admitted to ICU, the primary source of infection was bacteremia, followed by pneumonia. The use of vancomycin loading dose between the two groups was similar. The mean maintenance dose was 24.3 (± 11.1) mg/kg/day in the early group compared with 28.1(± 13.6) mg/kg/day in the late group (Table 1). The most common bacteria detected was Staphylococcus aureus (Table 2).

**Table 1:** Baseline characteristics

Variables	Before propensity score (PS) matching				After propensity score (PS) matching			
	Overall (326)	Early (110)	Late (216)	P-value	Overall (210)	Early (105)	Late (105)	P-value
Age (years), Mean (SD)	57.5 (20.17)	66.9 (16.55)	52.7 (20.19)	< .0001*	58.5 (18.41)	66.8 (16.65)	50.2 (16.29)	< .0001*
Male, n (%)	234 (72.0)	68 (62.4)	166 (76.9)	0.0061^^	147 (70.3)	66 (63.5)	81 (77.1)	0.0304^^
Weight (kg), Mean (SD)	71.6 (20.47)	72.7 (22.87)	71.0 (19.17)	0.8841^	72.3 (22.18)	72.8 (23.17)	71.9 (21.27)	0.9928^
Body mass Index, Mean (SD)	27.2 (16.77)	29.6 (27.10)	26.0 (7.13)	0.1379^	28.0 (20.42)	29.8 (27.72)	26.3 (8.16)	0.3033^
**Admission category**								
Medical, n (%)	179 (55.1)	78 (71.6)	101 (46.8)	0.0018^^	124 (59.3)	75 (72.1)	49 (46.7)	0.0054**
Surgical, n (%)	44 (13.5)	9 (8.3)	35 (16.2)		28 (13.4)	7 (6.7)	21 (20.0)	
Trauma, n (%)	47 (14.5)	9 (8.3)	38 (17.6)		26 (12.4)	9 (8.7)	17 (16.2)	
Burn, n (%)	8 (2.5)	1 (0.9)	7 (3.2)		3 (1.4)	1 (1.0)	2 (1.9)	
Neuro, n (%)	35 (10.8)	8 (7.3)	27 (12.5)		21 (10.0)	8 (7.7)	13 (12.4)	
Transplant, n (%)	12 (3.7)	4 (3.7)	8 (3.7)		7 (3.3)	4 (3.8)	3 (2.9)	
**Comorbidity**								
Diabetes mellitus, n (%)	142 (49.1)	62 (64.6)	80 (41.5)	0.0002^^	93 (50.5)	59 (64.8)	34 (36.6)	0.0001^^
Hypertension, n (%)	154 (52.0)	66 (66.0)	88 (44.9)	0.0006^^	102 (53.7)	62 (65.3)	40 (42.1)	0.0014^^
Chronic Liver disease, n (%)	27 (10.3)	7 (8.2)	20 (11.2)	0.4533^^	14 (8.6)	7 (8.8)	7 (8.4)	0.9426^^
Heart failure, n (%)	48 (17.8)	28 (30.4)	20 (11.2)	< 0.0001^^	37 (21.5)	27 (31.0)	10 (11.8)	0.0021^^
Cerebrovascular accident, n (%)	31 (11.4)	16 (16.8)	15 (8.5)	0.0384^^	26 (14.7)	16 (17.8)	10 (11.5)	0.2378^^
Chronic obstructive pulmonary disease (COPD), n (%)	16 (6.2)	8 (9.3)	8 (4.7)	0.1442^^	13 (7.9)	8 (9.9)	5 (6.0)	0.3612^^
**Severity score within 24 h of ICU admission**								
Modified Sequential Organ Failure Assessment (mSOFA), Median (Q1, Q3)	5.5 (4.00, 7.00)	5.0 (4.00, 8.00)	6.0 (4.00, 7.00)	0.9329^	5.0 (4.00, 7.00)	5.0 (4.00, 8.00)	5.0 (4.00, 7.00)	0.6435^
APACHE II Score, Median (Q1, Q3)	14.5 (9.00, 20.00)	15.0 (11.00, 21.00)	14.0 (9.00, 20.00)	0.2706^	15.0 (11.00, 21.00)	15.0 (11.00, 21.00)	13.0 (9.00, 20.00)	0.2249*
**Baseline within 24 h of ICU admission**								
Admission Glasgow Coma Scale (GCS), Median (Q1, Q3)	13.0 (7.00, 15.00)	11.0 (6.00, 15.00)	14.0 (7.00, 15.00)	0.2239^	13.0 (8.00, 15.00)	11.0 (6.00, 15.00)	14.0 (9.00, 15.00)	0.0606^
Mechanical ventilation within 24 h of ICU admission, n (%)	143 (44.3)	42 (38.9)	101 (47.0)	0.3663**	90 (43.5)	42 (40.8)	48 (46.2)	0.3091**
AKI within 24 h of ICU admission, n (%)	47 (14.5)	14 (12.8)	33 (15.3)	0.5559^^	40 (19.2)	20 (19.2)	20 (19.2)	> 0.9999^^
Blood glucose level (BGL) (mmol/L), Mean (SD)	10.2 (5.18)	10.5 (4.87)	10.0 (5.40)	0.2967^	10.7 (4.95)	10.6 (4.85)	10.8 (5.17)	0.8611^
Lactic acid (mmol/L), Mean (SD)	2.7 (2.40)	2.4 (1.69)	2.9 (2.70)	0.7442^	2.7 (2.26)	2.4 (1.71)	2.9 (2.69)	0.5299^
Serum creatinine (μmol/L), Mean (SD)	104.0 (73.53)	114.6 (77.44)	98.6 (71.07)	0.0162^	105.8 (70.31)	114.7 (78.28)	97.0 (60.49)	0.0586^
Blood urea nitrogen (BUN), Mean (SD)	8.5 (6.49)	9.8 (6.69)	7.9 (6.31)	0.0020^	8.6 (6.17)	9.5 (6.35)	7.6 (5.87)	0.0070^
Hematocrit, Mean (SD)	0.4 (0.08)	0.3 (0.09)	0.4 (0.08)	0.1298^	0.3 (0.09)	0.3 (0.09)	0.4 (0.09)	0.2032^
Albumin (g/L), Mean (SD)	30.8 (6.19)	29.5 (6.14)	31.4 (6.12)	0.0096*	30.7 (6.27)	29.5 (6.14)	31.8 (6.21)	0.0071*
Platelets count (1000 × 10^6/L), Mean (SD)	243.7 (141.00)	256.9 (148.17)	237.1 (137.20)	0.2522^	244.7 (148.49)	256.2 (148.29)	233.4 (148.52)	0.1837^
Alanine transaminase (ALT), Mean (SD)	68.4 (142.17)	46.1 (97.51)	79.2 (158.46)	0.0887^	61.7 (128.95)	47.0 (98.51)	75.9 (151.96)	0.2515^
Aspartate aminotransferase (AST), Mean (SD)	94.1 (232.29)	90.9 (314.56)	95.7 (178.18)	0.0537^	101.3 (271.91)	92.5 (317.86)	109.9 (219.27)	0.0564^
International normalized ratio (INR), Mean (SD)	1.3 (0.53)	1.4 (0.65)	1.3 (0.46)	0.5287^	1.3 (0.60)	1.4 (0.66)	1.3 (0.55)	0.8566^
Total Bilirubin, Mean (SD)	37.5 (86.08)	33.8 (72.26)	39.5 (92.83)	0.1594^	34.0 (68.26)	33.8 (72.73)	34.2 (63.84)	0.1658^
White blood cells (WBCs), Mean (SD)	13.0 (8.17)	12.5 (7.80)	13.3 (8.36)	0.4028^	13.0 (7.72)	12.6 (7.96)	13.4 (7.50)	0.2366^
Maximum temperature (T max)), Mean (SD)	37.8 (3.14)	37.9 (3.99)	37.8 (2.62)	0.2355^	37.7 (2.92)	37.9 (4.09)	37.6 (0.80)	0.3100^
**Vancomycin**								
Loading dose, n (%)	43 (13.2)	15 (13.8)	28 (13.0)	0.8410^^	27 (12.9)	14 (13.5)	13 (12.4)	0.8159^^
Maintenance dose (mg/kg/day), Mean (SD)	25.9 (12.16)	24.2 (11.10)	26.8 (12.62)	0.0909^	26.2 (12.58)	24.3 (11.19)	28.1 (13.65)	0.0524^
Initial level of vancomycin trough, Mean (SD)	8.1 (3.23)	11.7 (1.18)	6.3 (2.23)	< .0001*	9.0 (3.23)	11.7 (1.17)	6.3 (2.19)	< .0001*

^*^T Test/^Wilcoxon rank sum test is used to calculate the P-value

^^Chi square/** Fisher’s Exact teat is used to calculate P-value

**Table 2:** Source of infection and microorganism (s) after PS matching

	Early	Late	P-value^^
**Sources of gram-positive infection, n (%)**			
Bacteremia	56 (53.3)	55 (52.3)	0 .89
Pneumonia	29 (27.9)	35 (33.3)	0.37
Skin/wound infection (s)	13 (12.3)	12 (11.4)	0.83
Other source of infection	13 (12.3)	11 (10.5)	0.66
**Gram-positive organism, n (%)**			
*Staphylococcus aureus*	30 (28.8)	26 (24.8)	0.29
*Staphylococcus* (Non-aureus)	29 (27.9)	31 (29.5)	
*Streptococcus* spp.	4 (3.8)	11 (10.5)	
*Enterococcus* spp.	20 (19.2)	12 (11.4)	
Others	1 (1.0)	2 (1.9)	
**Concomitant gram-negative infection, n (%)**	31 (29.5)	22 (20.1)	0.15

^^Chi-square test is used to calculate the P-value

### Demographic and clinical characteristics

Characteristics of the patients are presented in Table 1. The majority of patients in both arms were men, and the mean age for patients was 66.8 and 50.2 years old in the early and late groups, respectively. After PS matching, comorbidities such as diabetes mellitus, hypertension, and heart failure were more prevalent in the early group. In addition, more medical patients were included in the early group than the late group; also, late group  have higher albumin levels and lower BUN. The median APACHE II score was 15 in the early groups compared to 13 in the late group (p = 0.22). Also, the median *modified sequential organ failure assessment* (*mSOFA*) score was 5 in both groups, which was not statistically significant (p = 0.64) after PS matching. Moreover, no significant differences were observed between the two groups in mechanical ventilation and AKI status within 24 h of ICU admission (Table 1).

### Primary outcome

In the crude analysis, 22 patients (23.2%) who attained therapeutic vancomycin levels early died within 30 days, compared to 25 patients (26.6%) in the late group (p = 0.58). Thirty-day mortality was higher in the late group compared to patients who reached early therapeutic trough levels in multivariable Cox proportional hazards regression analysis (HR: 2.54; 95 %CI [1.24–5.22]; p = 0.01) (Table 3).

**Table 3:** Regression analysis for the outcomes after PS matching

Outcomes	Early	Late	P-value	Hazard ratio (95%CI)	P-value$
30-day mortality, n (%)	22 (23.2)	25 (26.6)	0.5846^^	2.54 (1.24, 5.22)	0.01
				**Odds ratio (95%CI)**	P-value$*
Developing of vancomycin resistance organism (s) (e.g. (VRSA, VISA), n (%)	3 (2.9)	3 (2.9)	0.9905**	4.28 (0.47, 38.8)	0.19
Eradication of microorganism within 4–5 days of vancomycin initiation, n (%)	72 (69.2)	70 (66.7)	0.6913^^	1.49 (0.75, 2.96)	0.25
Vancomycin induced acute kidney injury, n (%)	13 (12.5)	19 (18.1)	0.2614^^	2.59 (1.01, 6.65)	0.04
				**Beta coefficient (Estimates) (95%CI)**	P-value $**
ICU length of stay (Days), Median (Q1, Q3) **&**	15.0 (9.00, 27.00)	13.0 (4.00, 24.00)	0.1802^	− 0.27 (− 0.58, 0.05)	0.10
Hospital length of stay (Days), Median (Q1, Q3) **&**	26.0 (14.00, 42.00)	24.0 (15.00, 44.00)	0.8722^	0.10 (− 0.22, 0.43)	0.54

^ Wilcoxon rank sum test is used to calculate the P-value

^^Chi-square test is used to calculate the P-value

**Fisher Exact test is used to calculate the P-value

^&^Denominator is patients who survived

^$^Cox proportional hazards regression analysis is used to calculate hazard ratio (HR) and p-value

^$*^Multivariate logistic regression analysis is used to calculate Odds ratio and p-value

^$**^Generalized linear model is used to calculate beta coefficient (estimates) and p-value

### Secondary outcome (s)

Thirteen patients (12.5%) in the early achievement group developed AKI compared to nineteen patients (18.1%) in the late group (p = 0.26) (Table 3). Despite the similar use of nephrotoxic medications between the two groups (Table 4), the late group had a higher odds of AKI (OR = 2.59; 95% CI [1.01–6.65]; p = 0.04). The mean time to develop AKI after vancomycin initiation was 2.4 (± 2.64) days in the late group. Among the 32 patients who developed acute kidney injury in both groups, eight patients required dialysis during ICU stay, of which three patients in the early group compared to five patients in the late group (Table 3).

**Table 4:** Concomitant use with other nephrotoxic medications

Nephrotoxic Medication (s)	Early	Late	P-value
Flucloxacillin IV, n (%)	1 (1.3)	2 (2.6)	0.5754**
Amikacin IV, n (%)	7 (9.2)	5 (6.5)	0.5320^^
Gentamicin IV, n (%)	12 (15.6)	18 (21.7)	0.3231^^
Colistin IV, n (%)	9 (11.8)	10 (12.8)	0.8536^^
Piperacillin/tazobactam IV, n (%)	63 (63.6)	68 (66.0)	0.7229^^
Sulfamethoxazole/trimethoprim IV, n (%)	3 (4.0)	9 (11.1)	0.0958^^
Furosemide IV, n (%)	61 (59.8)	52 (55.3)	0.5255^^
IV Contrast, n (%)	35 (33.7)	40 (38.5)	0.4703^^

^^Chi-square test is used to calculate the P-value

**Fisher Exact test is used to calculate the P-value

Patients who attained therapeutic vancomycin levels after 48 h have a higher odds of developing vancomycin resistance organism (s) (e.g. (VRSA, VISA); however, this finding did not reach statistical significance. Neither eradication of microorganisms within 4–5 days of vancomycin initiation nor the length of stay were statistically significant between the two groups.

## Discussion

Several studies have correlated optimizing pharmacokinetic-pharmacodynamics (PK-PD) with better clinical outcomes in Staphylococcus aureus related infections treated with IV vancomycin. Our results show that in a broad population of adult ICU patients with confirmed infection treated with vancomycin, the early attainment of therapeutic drug levels within 48 h was associated with a reduced risk of 30-day mortality. Compared to methicillin-susceptible S. aureus (MSSA), MRSA is independently associated with an increased risk of hospital mortality; thus, early attainment of MRSA treatments therapeutic levels is crucial [2, 4, 6, 12].

In our study, the early attainment of vancomycin therapeutic levels was associated with a statistically significant difference in 30 days mortality (HR: 2.54; p = 0.01). The significance of this outcome and its application might improve the clinical outcomes in critically ill patients, that has been highlighted in the recent surviving sepsis campaigns guidelines recommendation to improve the patient outcomes with early appropriate antibiotics therapy  [34]. The median time for vancomycin to reach a therapeutic level in previously reported data was three days, which is considered late in our study definition [13–16]. However, a prospective multicenter study that validated the vancomycin consensus guideline nomogram published in 2009 had a median time of two days [17]. Many strategies are suggested to achieve earlier trough levels using continuous vancomycin infusion [18–20]. Cardile et al. implemented a vancomycin therapeutic drug monitoring (TDM) program to reduce time to target trough attainment and evaluate its impact on clinical outcomes in an observational pre- and post- intervention study. The study found that patients on the TDM group were discharged earlier compared to the control group (7 vs. 14 days, respectively, p = 0.01), required shorter time to clinical stability (4 vs. 8 days, respectively, p = 0.02), and no difference in mortality between the groups [35].

Our results showed no statistically significant difference in vancomycin-associated resistance patterns in the early group compared to the late group. However, considering the relatively small number of included patients in our analysis and the fact that pathogen-specific MICs were not reported in our study are possible contributors in limiting our finding. Previous studies have reported an increase in the risk of developing resistance patterns of vancomycin with subtherapeutic vancomycin levels (< 10 mg/L) and inability to reach an appropriate minimum inhibitory concentration (MIC) to optimize PK-PD targets [21, 22].

MRSA related infections is associated with significant morbidity and mortality with limited therapeutic options, emergence of less susceptible strains, and safety concerns limiting the use of the available options [36]. Further, Hidayat et al., reported higher infection-related mortality in patients with MRSA infections with high MIC (24% vs. 10%) [23]. New modalities such as efflux pump are highly needed to further enhance antibiotics efficacy giving emergence of resistance through different mechanisms [37]. Using efflux pump inhibitors (e.g., NorA and P13CP) as an adjuvant to elevate the efficacy of antibiotics was studied and found to be effective against both Gram-positive and Gram-negative multi drug resistant (MDR) pathogens [38, 39].

Supratherapeutic vancomycin trough levels, among other factors such as concurrent nephrotoxic agents, concurrent vasopressor therapy, and undergone a procedure, are common risk factors for developing acute kidney injury (AKI) in critically ill patients [24–29]. We reported a higher odds of AKI in the patients who achieved the target level after 48 h (late group). Higher rates of AKIs were reported in studies that applied the AKIN criteria for nephrotoxicity as it reached 35%–37% when using intermittent vancomycin infusions compared to our study (21–25%) [24–26]. A recent systematic review suggested a higher risk of AKI with the co-administration of piperacillin/tazobactam; however, in our data, the use of other concomitant nephrotoxic medications was similar between the two groups (Table 4) [30].

Our study has several limitations: first, the retrospective and single center study design. Secondly, our population's heterogeneity (medical, surgical, burn, and trauma ICU patients) might have affected our outcomes. Thirdly, we reported mortality for a small sample size study with many potential confounders; however, none of these variables was shown to impact our outcome based on our univariate regression analysis. Additionally, we were able to report nephrotoxic medication co-administration and contrast use. We acknowledge the revised consensus guidelines for vancomycin monitoring in MRSA infections to use individualized target area under the curve (AUC) over MIC, however many clinicians are still using the previous recommendation for targeting trough levels of 15–20 mg/L in daily practice [7, 8]. Adopting AUC/MIC consensus guidelines in the developed countries may need more time and education [7]. Additionally, taking ARC impact on the clinical outcomes might be of value to address because of its relationship with timing to achieve the therapeutic level [31–33]. Future studies are needed to confirm our findings.

## Conclusion

Early attainment of therapeutic levels of vancomycin within 48 h of initiation may be associated with plausible survival benefits. More studies are needed to provide an insight into these correlations.

## Data Availability

The datasets used and/or analyzed during the current study are available from corresponding author on reasonable request.

## Abbreviations

ICUs: Intensive care units
AKI: Acute kidney injury
LOS: Length of stay
MRSA: Methicillin-resistant Staphylococcus aureus
Pk-PD: Pharmacokinetic-pharmacodynamic
TDM: Therapeutic drug monitoring
AUC: Area under the curve
ADR: Adverse drug reaction
IDSA: Infectious diseases society of America
